# Electroacupuncture improves myocardial ischemia injury via activation of adenosine receptors

**DOI:** 10.1007/s11302-020-09704-3

**Published:** 2020-07-06

**Authors:** Yulan Ren, Zhihan Chen, Rui Wang, Yang Yu, Dehua Li, Yonggang He

**Affiliations:** 1 School of Chinese Classics, Chengdu University of Traditional Chinese Medicine, No. 37, Shierqiao Road, Jinniu District, Chengdu, Sichuan China; 2 School of Acupuncture Moxibustion and Tuina, Chengdu University of Traditional Chinese Medicine, Chengdu, Sichuan China; 3 Clinical Medical College, Chengdu University of Traditional Chinese Medicine, Chengdu, Sichuan China

**Keywords:** Acupuncture, Myocardial ischemia, A1 adenosine receptor, A2a adenosine receptor, A2b adenosine receptor

## Abstract

Electroacupuncture (EA) can improve myocardial ischemia (MI) injury; nevertheless, the mechanism is not entirely clear. And there were disagreements about whether the effect of EA at acupoint in disease-affected meridian is better than EA at acupoint in non-affected meridian and sham acupoint. Here, we showed that the effect of EA at Neiguan (PC6) is better than EA at Hegu (LI4) and sham acupoint in affecting RPP and ECG, increasing ATP and ADO production, decreasing AMP production, and upregulating the mRNA expression levels of A1AR, A2aAR, and A2bAR; knockdown of A1AR or A2bAR reversed the effect of EA at PC6 in alleviating MI injury; knockdown of A2aAR had no influence on the cardiac protection of EA at PC6; thus, the cardioprotective effect of EA at PC6 needs A1AR and A2bAR, instead of A2aAR; considering that the cardio protection of adenosine receptor needs activation of other adenosine receptors, one of the reasons may be that after silence of A1AR or A2bAR, EA at PC6 could not impact the expression levels of the other two adenosine receptors, and after silence of A2aAR, EA at PC6 could impact the expression levels of A1AR and A2bAR. These results suggested that EA at PC6 may be a potential and effective treatment for MI by activation of A1AR and A2bAR.

## Introduction

Myocardial ischemia (MI) is one of heart conditions caused by lack of coronary blood flow [1], which is the primary cause of ischemic heart disease (IHD). The “The top 10 causes of death” indicated that IHD is one of the biggest killers in the world, accounting for around 9 million death in 2016 [2]. “Guideline on the Assessment and Management of Cardiovascular Risk in China,” which was published in 2019, showed that cardiovascular disease is the leading cause of death and burden of disease in China, and over 1 million people died of coronary heart disease [3]. In addition, IHD burden the economy [3, 4]. Currently, the primary therapeutic methods include percutaneous coronary intervention, coronary artery bypass grafting, and medical therapy [5–11]. On account of the efficacy and safety of acupuncture, increasing patients are willing to receive acupuncture treatment. There is long history of application of acupuncture in improving MI. Previous studies indicated that acupuncture is effective in alleviating MI [12–15], and Neiguan (PC6) is the most frequently used acupoint [16]. The effect of acupuncture in MI was verified; nevertheless, there are different results of the effect of acupoint in disease-affected meridian, acupoint in non-affected meridian, and sham acupoint [12, 17, 18]. Previous animal experiments were conducted to reveal mechanism of acupuncture in improving MI [13, 19, 20]. Some experiments showed that acupuncture could impact the express level of A1 adenosine receptor (A1AR) or A2a adenosine receptor (A2aAR) or A2b adenosine receptor (A2bAR) [21, 22]. However, it cannot verify that the effect of acupuncture in MI depends on A1AR, A2aAR, and A2bAR. Thus, we hypothesized that acupoint in disease-affected meridian might be more effective than acupoint in non-affected meridian and sham acupoint; the effect of acupuncture at acupoint in disease-affected meridian in improving MI may depend on A1AR or A2aAR or A2bAR. To test these hypotheses, this experiment was conducted to evaluate (a) the differences in the effect of acupoint in disease-affected meridian (PC6), acupoint in non-affected meridian (Hegu (LI4)), and sham acupoint; (b) the roles of A1AR, A2aAR, and A2bAR in the effect of acupuncture at PC6; and (c) the possible reason for the results that the effect of acupuncture at PC6 depends on one of adenosine receptors.

## Materials and methods

### Subjects

Male and female Sprague-Dawley (SD) rats (Charles River, Beijing, China), weighing 200–220 g, received ad libitum food and water, and were housed in a temperature-controlled room (25 ± 3 °C) under a 12-h light/dark cycle. This study was conducted in accordance with the Guide for the Care and Use of Laboratory Animals (National Academies Press) and was approved by ethical committee of Chengdu University of Traditional Chinese Medicine (no. 2014–03).

### Reagents

Recombinant virus plasmid and the carriers of auxiliary packaging original plasmids were purchased from Shanghai Genechem Co., Ltd. (Shanghai, China). Adora1, adora2a, and adora2b mRNA in situ hybrization kits were purchased from Boster Biological Technology Co., Ltd. (CA, USA). 293 T cell was purchased from Cell Resource Center of Shanghai Institutes for Biological Sciences (Shanghai, China).

### Grouping

After 1-week acclimatization, 140 rats were randomly divided equally into 14 groups, namely normal control (NC) group, sham operation (SO) group, model (M) group, Neiguan (N) group, Hegu (H) group, sham acupoint (SA) group, negative lentivirus infected (NLI) group, A1AR lentivirus-infected (A1LI) group, A2aAR lentivirus-infected (A2aLI) group, A2bAR lentivirus-infected (A2bLI) group, negative lentivirus-infected Neiguan (NLIN) group, A1AR lentivirus-infected Neiguan (A1LIN) group, A2aAR lentivirus-infected Neiguan (A2aLIN) group, and A2bAR lentivirus-infected Neiguan (A2bLIN) group. In NC group, rats received no operation; in SO group, rats were only threaded, but not ligated left anterior descending coronary artery (LADCA); in M group, rats received ligation of LADCA; in N group, rats received ligation of LADCA and electroacupuncture (EA) treatment at bilateral PC6; in H group, rats received ligation of LADCA and EA treatment at bilateral LI4; in SA group, rats received ligation of LADCA and EA treatment at bilateral sham acupoint; in NLI, A1LI, A2aLI, and A2bLI groups, rats received corresponding lentivirus injection and ligation of LADCA; in NLIN, A1LIN, A2aLIN, and A2bLIN groups, rats received corresponding lentivirus injection, ligation of LADCA, and EA treatment at bilateral PC6.

### MI injury

**Fig. 1:**
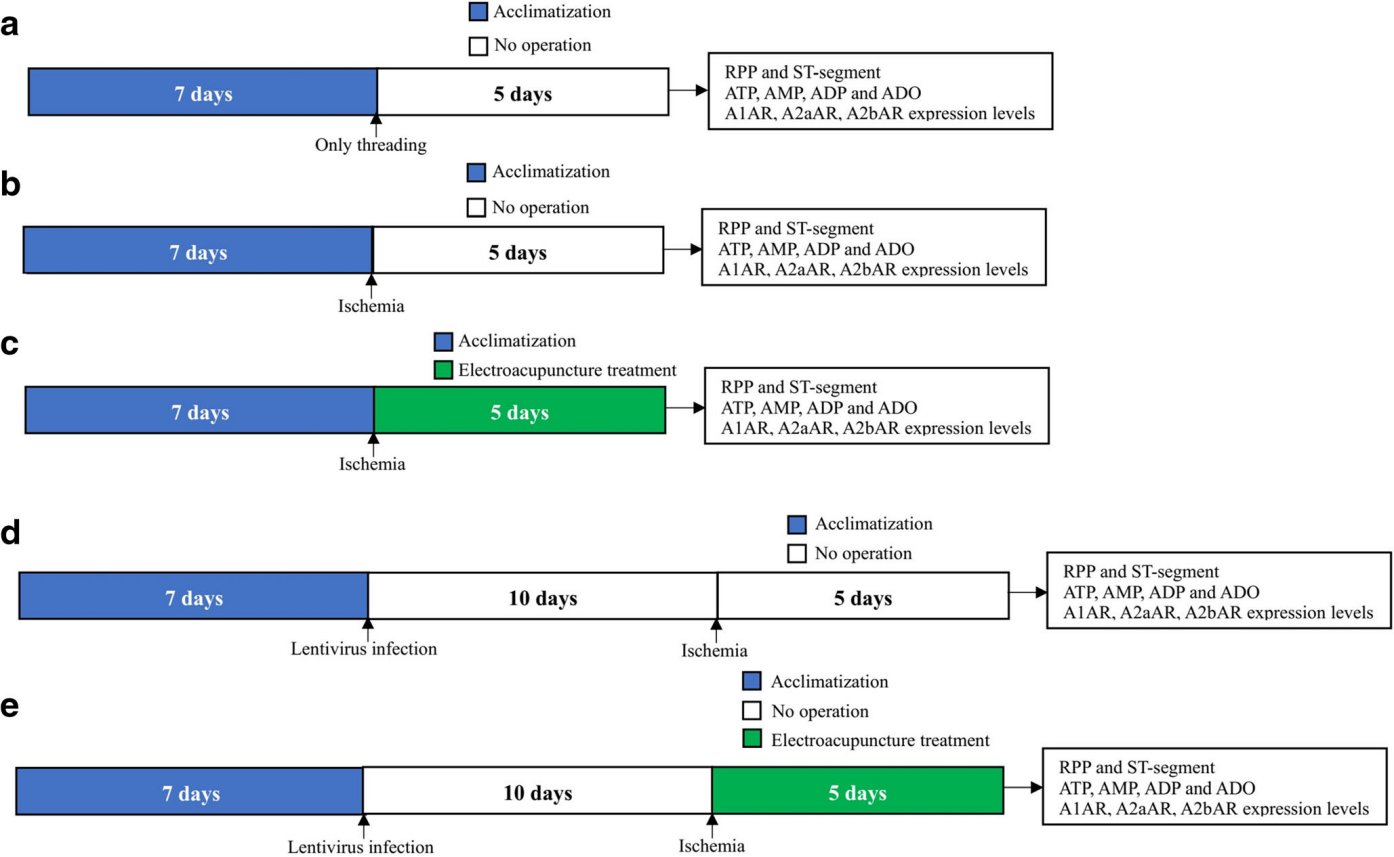
Flow chart of the study protocol. **a** SO group. **b** M group. **c** N, H, and SA group. **d** NLI, A1LI, A2aLI, and A2bLI group. **e** NLIN, A1LIN, A2aLIN, and A2bLIN group

The rats were anesthetized with 10% chloral hydrate (0.4 ml/100 g IP) and were positioned on an operating table for small animals (Fig. 1). After a thoracotomy performed in the fourth intercostal space, the LADCA was ligated 2 to 3 mm from its origin between the pulmonary artery conus and the left atrium with 6–0 silk. Occlusion of LADCA was verified by regional color change in the myocardial surface local and distal to the ligation, and change in electrocardiography (ECG). For M group, the LADCA of rats were only threaded with 6–0 silk.

### Lentivirus infection

The rats were anesthetized by intraperitoneal injection of chloral hydrate and received above thoracotomy. After exposure of heart, 50 μl corresponding lentivirus solution (1 × 10^7^ TU/100 μl) was injected into left ventricle anterior wall at four different points. Finally, the chest was closed.

### EA treatment

The rats received EA treatment a day after MI operation. Each EA treatment lasted 20 min; the rats received 5 times of treatment once every day for 5 consecutive days. Once-off stainless steel acupuncture needle (Suzhou Hualun Medical Appliance Co., Ltd., Jiangsu, China) was inserted to a depth of 1–2 mm. The acupuncture needle was connected to the HANS Acupuncture Point Nerve Stimulator (Nanjing Jisheng Medical Technology Co., Ltd., Jiangsu, China). The frequency was set at 2/100 Hz, and the intensity of stimulation was 0.1–1 mA. The procedures of all EA groups (N, H, SA, NLIN, A1LIN, A2aLIN, A2bLIN groups) were the same. The location of acupoints, which refers to previous literatures [23, 24], is as following: (1) PC6: on the anteromedial aspect of the forelimb, between the ulna and the radius, 3 mm proximal to the wrist joints; (2) LI6: on forelimb, between the first metacarpal bone and the second metacarpal bone; and (3) sham acupoint: on the dorsal aspect of the forelimb, the spatia between the third metatarsal bone and the fourth metatarsal bone.

### Determination of rate pressure product and ST segment

Left ventricular developed pressure (LVDP) and heart rate (HR) obtained by Data Acquisition System (iWorx, IL, USA) were used to calculate rate pressure product (RPP), according to the previously described method [25]: RPP = LVDP × HR. Data Acquisition System was also adopted to record ECG. All rats were calculated RPP and were recorded ECG before taking sample.

### High-performance liquid chromatography

HPLC was used to determine the production of ATP, AMP, ADP, and ADO in myocardial tissue. The samples were determined by 2 mol/l HClO_4_ (20 μl/mg). After centrifugation at 4 °C and 20,000×*g* for 10 min, aliquots of the supernatant were collected. To collect supernatant to regulate PH value to 6.5–7.0, 1 mol/l K_2_CO_3_ was added. After blending, the mixture was stood on ice for 5 min. After centrifugation at 4 °C and 20,000×*g* for 10 min, the supernatant was collected for HPLC determination of ATP, AMP, ADP, and ADO concentrations. The chromatographic conditions were as follows: (1) chromatographic column: hypersil C18, 5 μm, 4.6 × 200 mm; (2) mobile phase: 0.2 mol/l phosphate potassium salt buffer (pH 6.0); and (3) flow rate 0.9 ml/min. Ultraviolet-visible detector (Dalian Elite Analytical instrument Co., Ltd., Liaoning, China) was adopted to carry out chromatographic analysis.

### In situ hybrization

Cardiac tissue was fixed in 4% paraformaldehyde overnight and was soaked in phosphate buffer solution (PBS) 4–5 times for 5 min. Cardiac tissue was put into 30% sucrose/0.1 M PBS. Two days later, cardiac slice was soaked in 0.1 M PBS (once for 5 min), 0.1 M glycine/0.1 M PBS (once for 5 min), 0.3% Triton X-100/0.1 M PBS (once for 10 min), and 0.1 M PBS (three times for 5 min). Fifty microliter proteinase was added to incubate at 37 °C for 30 min. Cardiac slice was soaked in 4% paraformaldehyde for 5 min and was washed by 0.1 M PBS twice for 5 min. Cardiac slice was put into 0.25% acetic anhydride/0.1 M triethanolamine for 10 min. Cardiac slice was pre-hybridized by pre-hybridization solution at 42 °C for 30 min and was hybridized by hybridization solution at 42 °C overnight. Four times SSC, 2× SSC, 1× SSC, 0.5× SSC, 0.2× SSC, 0.2× SSC/0.1 M PBS, 0.05 M PBS were, respectively, used to wash cardiac slice. Then, cardiac slice was coated by 3% BSA/0.05 M PBS at 37 °C for 30 min. Anti-digoxin/antiserum alkaline phosphatase was added to cardiac slice, and cardiac slice was, respectively, washed by 0.05 M PBS, TSM1, and TSM2. ImagePro Plus 6.0 software (Media Cybernetics, Inc., MD, the USA) was adopted to analyze results.

### Design and construction of lentivirus RNA interference vector

Short hairpin RNA (shRNA) interference sequences were designed for A1AR, A2aAR, and A2bAR (accession no. NM_017155, NM_053294, and NM_017161) to construct recombinant shuttle plasmids and packaging plasmids (pFU-GW-RNAi). The target sequences for A1AR, A2aAR, A2bAR, and negative control are as follows: 5′-CTTCTTTGCGTTCGTGTTA-3′, 5′-GATTTGGAATGACCACTTC-3′, 5′-GTGTCTCTTTGAGAACGTA-3′, 5′-TTCTCCGAACGTGTCACGT-3′. 293 T cells were transfected by sequenced plasmids and transfection reagent RNA-mate. The supernatants with lentivirus particles were concentrated and purified.

### Statistical analysis

Statistical analysis was made using SPSS 26.0 software (IBM, NY, USA). All data were expressed as mean ± SD and were analyzed using one-way ANOVA with post hoc Tukey tests or Games-Howell tests. Differences were considered statistically significant when *P* values were less than 0.05.

## Results

### Effects of EA on RPP and ST segment

**Fig. 2:**
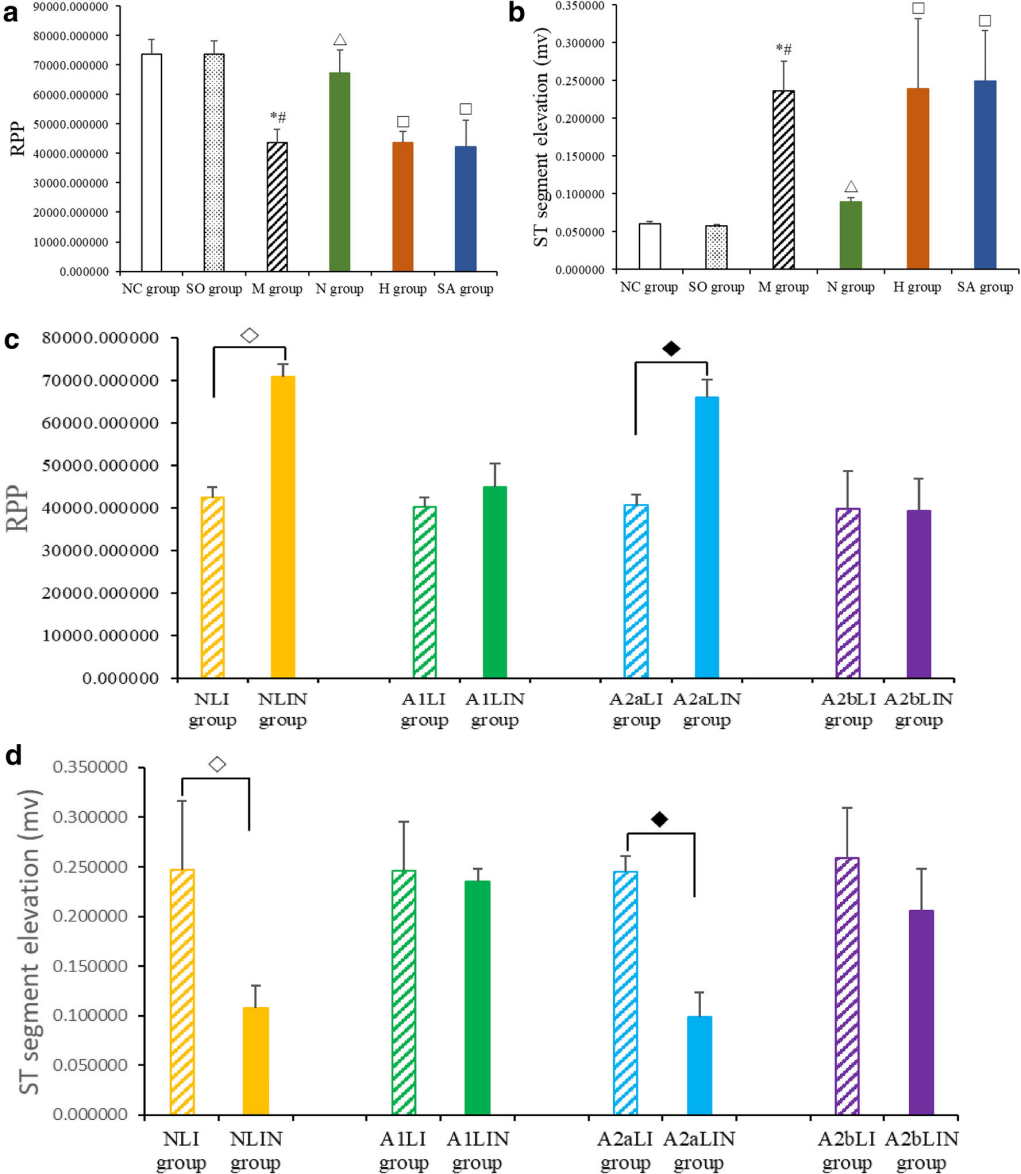
Effects of EA on RPP and ST segment. **a**, **c** Rate pressure product (RPP) was calculated by left ventricular developed pressure (LVDP) and heart rate (HR). EKG was recorded from II limb leads with recorder speed 50 ms/div, and ST segment alterations were dedicated in **b** and **d** after 5 days followed by surgical procedure. Data are presented as mean ± SD. *P < 0.01 versus NC group, #*P* < 0.01 versus SO group, △*P* < 0.01 versus M group, ◇*P* < 0.01 versus NLI group, ◆*P* < 0.01 versus A2aLI group

As shown in Fig. 1a, b, the value of RPP was significantly reduced and ST segment was significantly elevated in the M group compared with the NC group and the SO group (all *P* < 0.01); thus, MI injury model is successful. Compared with the M group, the value of RPP was significantly increased and ST segment was depressed in the N group (Fig. 2a, b, all *P* < 0.01) and the value of RPP and ST segment had no significant change in the H group and the SA group (Fig. 2a, b, all *P* > 0.05). Compared with the N group, the value of RPP was reduced and ST segment were elevated in the H group and the SA group (Fig. 2a, b, all *P* < 0.01).

In addition, compared with the NLI group, the value of RPP was increased and ST segment was depressed in the NLIN group (Fig. 2c, d, all *P* < 0.01); compared with the A1LI group, the value of RPP and ST segment in the NLIN group had no significant change (Fig. 2c, d, all *P* > 0.05); compared with the A2aLI group, the A2aLIN group had significantly increase the value of RPP and depress ST segment (Fig. 2c, d, all *P* < 0.01); compared with the A2bLI group, the A2bLIN group had no significant change in the value of RPP and ST segment (Fig. 2c, d, all *P* > 0.05).

### Effects of EA on ATP, AMP, ADP, and ADO concentrations

**Fig. 3:**
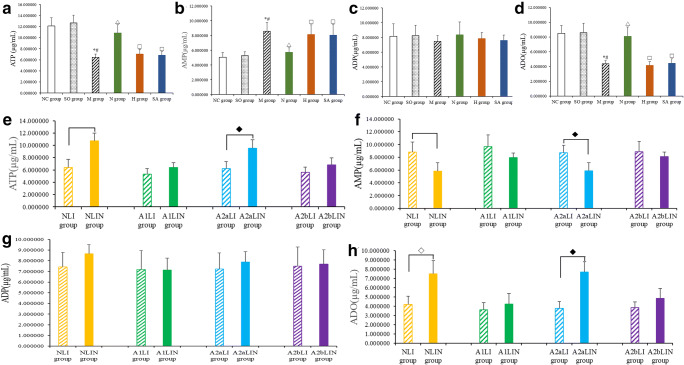
Effects of EA on ATP, AMP, ADP, and ADO concentrations. The ATP (**a**, **e**), AMP (**b**, **f**), ADP (**c**, **g**), and ADO (**d**, **h**) levels in the cardiac tissue. Data are presented as mean ± SD. *P < 0.01 versus NC group, #*P* < 0.01 versus SO group, △*P* < 0.01 versus M group, ◇*P* < 0.01 versus NLI group, ◆*P* < 0.01 versus A2aLI group

The results showed that MI injury caused decrease in the concentrations of ATP and ADO and increase in the concentration of AMP (Fig. 3a–d, all *P* < 0.01) and no change in the concentration of ADP (Fig. 3c, all *P* > 0.05) compared with the NC group and the SO group. EA treatment at PC6 significantly increased the concentrations of ATP and ADO, and decreased the concentration of AMP (Fig. 3a–d, all *P* < 0.01); however, EA treatment at PC6 did not significantly impacted the concentration of ADP (Fig. 3c, *P* > 0.05). EA treatment at LI6 and sham acupoint had no significant impact on the concentrations of ATP, AMP, ADP, and ADO (Fig. 3a–d, all *P* > 0.05). Compared with the H group and the SA group, the concentrations of ATP and ADO were increased, and the concentration of AMP was reduced by EA treatment at PC6 (Fig. 3a–d, all *P* < 0.01), and the concentration of ADP was not impacted (Fig. 3c, *P* > 0.05).

Figure 3e–h show that the concentrations of ATP and ADO have significantly risen and the concentration of AMP is decreased in the NLI group (all *P* < 0.01), and the concentration of ADP is not different from that of the NLI group (*P* > 0.05); the A1LI group and the A1LIN group have no statistical difference in the concentrations of ATP, AMP, ADP, and ADO (all *P* > 0.05); the concentrations of ATP and ADO are significantly increased, and the concentration of AMP is decreased (all *P* < 0.01), and the concentration of ADP is not changed (*P* > 0.05) in the A2aLIN group compared with the A2aLI group; the A2bLI group and the A2bLIN group also have no significant difference in the concentrations of ATP, AMP, ADP, and ADO (all *P* > 0.05).

### Effects of EA on the mRNA expression levels of A1AR, A2aAR, and A2bAR

**Fig. 4:**
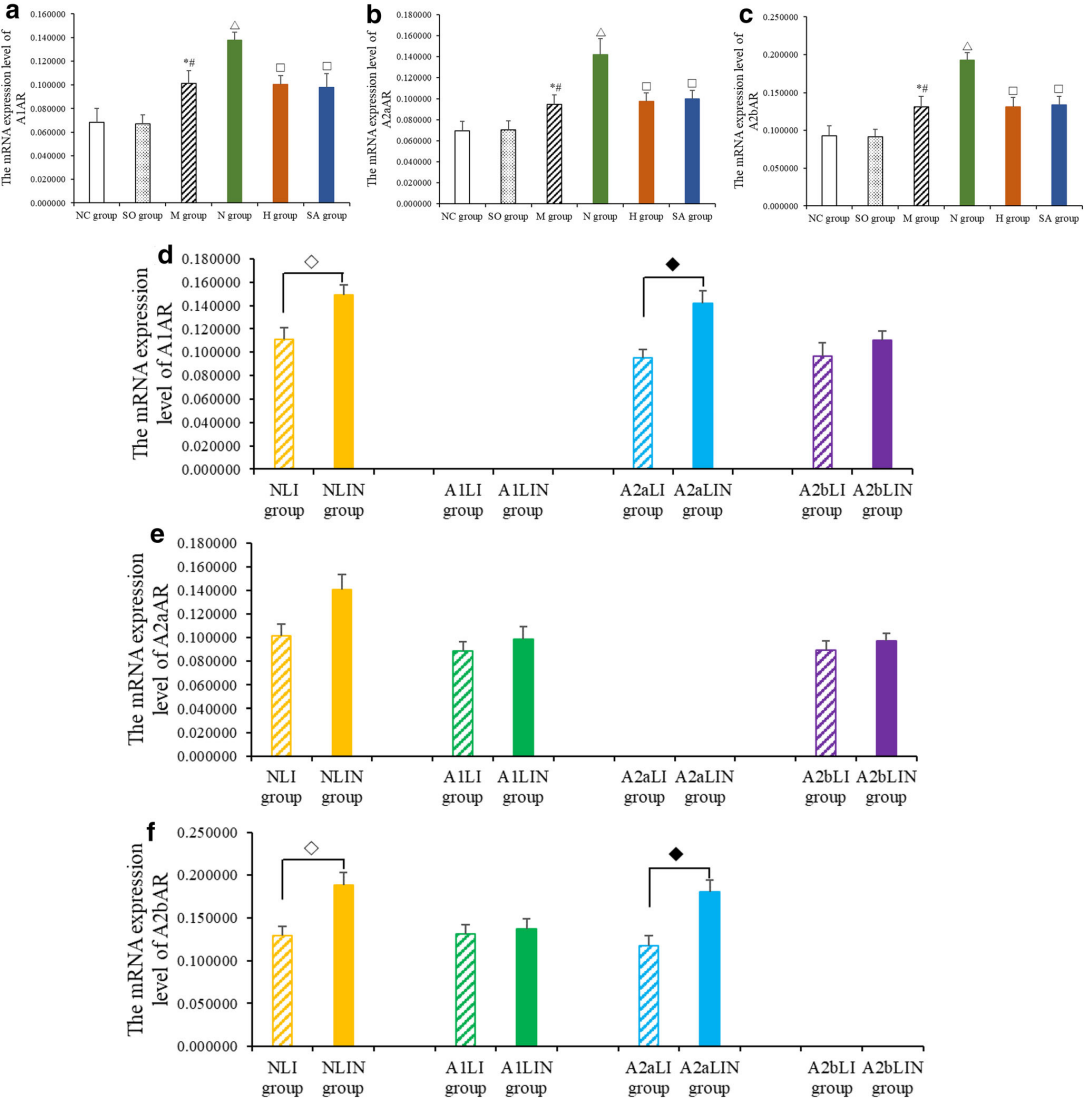
Effects of EA on the mRNA expression levels of A1AR, A2aAR, and A2bAR. The mRNA expression levels of A1AR (**a**, **d**), A2aAR (**b**, **e**), A2bAR (**c**, **f**) detected by in situ hybrization (ISH). Data are presented as mean ± SD. *P < 0.01 versus NC group, #*P* < 0.01 versus SO group, △*P* < 0.01 versus M group, ◇*P* < 0.01 versus NLI group, ◆*P* < 0.01 versus A2aLI group

Compared with the NC group and the SO group, the mRNA expression levels of A1AR, A2aAR, and A2bAR were significantly upregulated in the M group (Fig. 4a–c, all *P* < 0.01). EA treatment at PC6 could significantly upregulate the mRNA expression levels of A1AR, A2aAR, and A2bAR (Fig. 4a–c, all *P* < 0.01); however, EA treatment at LI6 and sham acupoint could not significantly upregulate the mRNA expression levels of A1AR, A2aAR, and A2bAR (Fig. 4a–c, all *P* > 0.05). The mRNA expression levels of A1AR, A2aAR, and A2bAR were higher in the N group than those of the H group and the SA group (Fig. 4a–c, all *P* < 0.01).

The mRNA expression levels of A1AR, A2aAR, and A2bAR in the NLIN group were higher than those of the NLI group (Fig. 4d–f, all *P* < 0.01); no significant differences were observed in the mRNA expression levels of A2aAR and A2bAR between the A1LI group and the A1LIN group (Fig. 4d–f, all *P* > 0.05); compared with the A2aLI group, the mRNA expression levels of A1AR and A2bAR were upregulated in the A2aLIN group (Fig. 4d–f, all *P* < 0.01); there was no statistical difference in the mRNA expression levels of A1AR and A2aAR between the A2bLI group and the A2bLIN group (Fig. 4d–f, all *P* > 0.05).

## Discussion

Our results indicated that (a) EA at PC6 has better effect than EA at LI6 and sham acupoint in affecting RPP and ECG, increasing ATP and ADO production, decreasing AMP production, and upregulating the expression levels of A1AR, A2aAR, and A2bAR; (b) A1AR and A2bAR are involved in EA-induced cardio protection in MI injury; and (c) silence of A1AR or A2bAR reverses the influence of EA at PC6 in upregulating the other two adenosine receptors and silence of A2aAR has no influence on the effect of EA at PC6 in upregulating A1AR and A2bAR.

MI induced depletion of ATP, and the decrease of ATP is due to inadequate rate of production of high-energy phosphate relative to the demand of the heart for energy [26]. ATP can be degraded to AMP and ADO [27, 28], and ADO can be degraded to inosine, trioxypurine, and so on [28]. Our results also showed that MI causes the reduction of ATP and ADO, and the increase of AMP. Previous studies indicated that recovery of myocardial function after MI is relevant to recovery of ATP [29], conversion of AMP to ADO has anti-inflammatory effect [30], and ADO has function of regulating inflammatory by binding to adenosine receptors [31], thus, it is speculated that the effect of EA at PC6 may be related to the regulating effect of EA at PC6 in ATP, AMP, and ADO.

Knockdown of A1AR or A2bAR abolished EA at PC6-induced functional protection via affecting RPP and ECG. Moreover, knockdown of A1AR and A2bAR reversed influences of ATP, AMP, and ADO production caused by EA at PC6. The cardio protection of adenosine receptor needs activating other adenosine receptors [32–35]. In our results, knockdown of A1AR or A2bAR reversed upregulation of the other two adenosine receptor expression levels and knockdown of A2aAR could not abolish the influence of EA at PC6 in upregulating the expression levels of A1AR and A2bAR. These observations showed that cardio protection of EA requires activating A1AR and A2bAR, but not A2aAR, and indicated one of reasons for these results.

Activating adenosine receptors can improve MI [36, 37]. For instance, activated A2aAR can reduce cardiac mast cell degranulation and protect ischemic myocardium [38]; A2bAR agonist has effect on improving MI injury, and this effect depends on activation of protein kinase C [37]. Previous experiment also detected the expression levels of A1AR, A2aAR, and A2bAR of MI rats after EA treatment [22]; both studies agreed that EA at PC6 could effectively upregulate the expression levels of A2aAR and A2bAR. On account of the differences in EA treatment protocol and detection method for the expression levels of adenosine receptors, there were disagreements about whether EA at PC6 could affect the expression level of A2aAR. Considering the upregulation of adenosine receptors is not equated with the activation of adenosine receptors, we knockdown the expression level of adenosine receptors. After silence of A1AR or A2bAR, EA at PC6 could not improve MI injury, and after silence of A2aAR, EA at PC6 still attenuate MI injury. Thus, the cardio protection of EA at PC6 needs A1AR and A2bAR.

The protective effect of A1AR, A2aAR, and A2bAR needs other adenosine receptors, namely only one adenosine receptor cannot play its role. A2aAR and A2bAR must be simultaneously activated for cardiac protection to occur [33]. The antagonism of A2aAR and A2bAR can block the protective effect of A1AR [34]. Other previous experiments also showed that the cardiac protection of A1AR needs the activation of A2aAR and A2bAR [32, 35]. In these results, after the silence of A1AR or A2bAR, EA at PC6 could not upregulate the expression levels of other two adenosine receptors, and after the silence of A2aAR, EA at PC6 still upregulate the expression levels of A1AR and A2bAR. It may be one of reasons why the effect of EA at PC6 in improving MI depends on A1AR and A2bAR, not A2aAR.

## Conclusion

In conclusion, this study indicated that EA at PC6 is effective in alleviating MI injury, EA at PC6 is more effective than EA at LI6 and sham acupoint; after silence of A1AR or A2bAR, EA at PC6 could not improve MI injury; thus, the effect of EA at PC6 may depend on A1AR and A2bAR. In addition, one of the reasons may be that after the silence of A1AR or A2bAR, EA at PC6 could not impact the expression levels of the other two adenosine receptors, and after the silence of A2aAR, EA at PC6 could impact the expression levels of A1AR and A2bAR.

## Data Availability

Not applicable.
